# Interpreting the Mechanism of Active Ingredients in Polygonati Rhizoma in Treating Depression by Combining Systemic Pharmacology and In Vitro Experiments

**DOI:** 10.3390/nu16081167

**Published:** 2024-04-14

**Authors:** Xin Wei, Dan Wang, Jiajia Liu, Qizhi Zhu, Ziming Xu, Jinzhe Niu, Weiping Xu

**Affiliations:** 1 Institute of Intelligent Machines, Hefei Institutes of Physical Science, Chinese Academy of Sciences, Hefei 230031, China; 2 Division of Life Sciences and Medicine, University of Science and Technology of China, Hefei 230026, China; 3 School of Food and Biological Engineering, Hefei University of Technology, Hefei 230601, China; 4 Anhui Provincial Key Laboratory of Tumor Immunotherapy and Nutrition Therapy, Hefei 230001, China

**Keywords:** Polygonati Rhizoma, depression, systemic pharmacology, DFV, inflammation

## Abstract

Polygonati Rhizoma (PR) has certain neuroprotective effects as a homology of medicine and food. In this study, systematic pharmacology, molecular docking, and in vitro experiments were integrated to verify the antidepressant active ingredients in PR and their mechanisms. A total of seven compounds in PR were found to be associated with 45 targets of depression. Preliminarily, DFV docking with cyclooxygenase 2 (COX2) showed good affinity. In vitro, DFV inhibited lipopolysaccharide (LPS)-induced inflammation of BV-2 cells, reversed amoeba-like morphological changes, and increased mitochondrial membrane potential. DFV reversed the malondialdehyde (MDA) overexpression and superoxide dismutase (SOD) expression inhibition in LPS-induced BV-2 cells and decreased interleukin-1β (IL-1β), tumor necrosis factor-α (TNF-α), and IL-6 mRNA expression levels in a dose-dependent manner. DFV inhibited both mRNA and protein expression levels of COX2 induced by LPS, and the activation of NACHT, LRR, and PYD domains-containing protein 3 (NLRP3) and caspase1 was suppressed, thus exerting an antidepressant effect. This study proves that DFV may be an important component basis for PR to play an antidepressant role.

## 1. Introduction

Depression is one of the most widespread and serious mental disorders globally, mainly manifested as despair, insomnia, and even suicide [1]. With the increased social pressures in life, the prevalence and incidence of depression continue to rise [2], especially in the background of the COVID-19 outbreak [3]. It is becoming a worldwide public health problem [4]. It is urgent to actively seek effective antidepressant drugs and elucidate their pharmacological mechanisms.

Polygonati Rhizoma (PR), Chinese name “Huangjing”, is a medicinal and dietary homolog of the *Polygonatum* Mill. in Liliaceae [5]. PR was first recorded in Shen Nong Ben Cao Jing (Eastern Han Dynasty, 25–220 AD) and has been applied as a traditional Chinese medicine (TCM) and nutritional supplement, with a history of more than 2000 years [6,7]. The theory of TCM points out that the key pathogenesis of depression is related to the shortcoming of five viscera and blood [8]. PR has the effects of nourishing the blood, heart, and liver, invigorating the spleen, tonifying the middle, moistening the lungs, and producing fluid. It has been used to treat depression in recent years with good effects [9]. Our previous studies found that saponins of PR effectively ameliorate the behavioral indicators of depression-model mice, reverse the abnormal expression of 5-hydroxytryptamine (5-HT) and other neurotransmitters in mice [10,11], and regulate the level of trace elements in depression-model mice [12]. Further, it has been proven to improve chronic stress-induced depression in rats through the 5-HT-related pathways [13,14]. However, due to the complexity of the components of Chinese herbal medicines, the mechanism of the specific compound has not yet been clearly explained by current studies. Researchers have proposed combining systemic pharmacology with molecular docking techniques to screen active compounds in complex components and predict their targets [15,16,17]. Delightedly, the researcher used these two techniques to successfully clarify the potential therapeutic effect of PR on COVID-19 and osteoporosis [5,18]. Researchers have combined systematic pharmacology and in vitro and in vivo experimental validation to elucidate the targeted kinase inhibitory activity of PR [19], and combined with metabolomics, to preliminarily reveal the key active ingredients of PR and its potential for treating Alzheimer’s disease (AD) [20]. This provides a feasible approach for exploring the mechanism of PR in treating depression.

**Figure 1:**
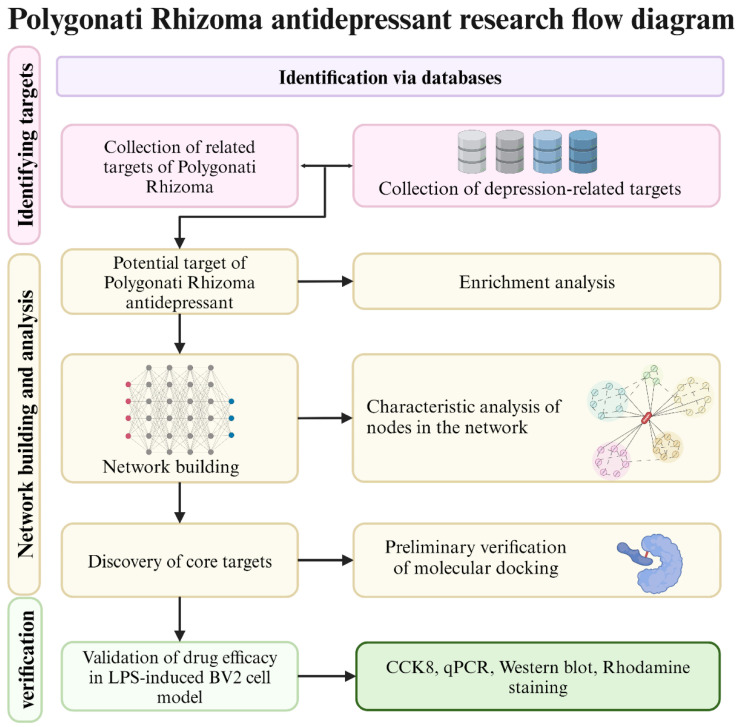
Flow chart of mechanism research of PR in treating depression (created with BioRender.com, accessed on 10 March 2024).

The microglia is a weighty member of the immune cells in the brain, and it participates in the immune defense and homeostasis maintenance of the systema nervosum centrale [21]. Lipopolysaccharide (LPS)-activated BV-2 microglia is a classic model commonly used to study the inflammatory state and interventional regulation of the nervous system in vitro [22,23,24]. In this study, we investigated the core chemicals and targets of PR in treating depression by integrating systemic pharmacology and molecular docking. Among them, the compound DFV associated with the core target, also known as Liquiritigenin or 7,4′-dihydroxyflavanone, has attracted our attention. DFV belongs to the flavonoid compounds in PR. Previous studies have shown that DFV has liver protection [25], anti-myocardial ischemia [26], anti-breast cancer [27], and bone protection effects [28,29]. In these studies, DFV demonstrated strong anti-inflammatory and antioxidant stress-resistance abilities. Based on the important roles of oxidative stress and inflammatory response in depression, as well as the progress of anti-inflammatory drugs in the treatment of depression, this study aims to explore the targets and molecular regulatory mechanisms of DFV antidepressant therapy. Previous researchers have explored the therapeutic effects and molecular mechanisms of DFV in a mice depression model in vivo and found that DFV alleviates depression-like behavior in mice by regulating the PI3K/Akt/mTOR-mediated brain-derived neurotrophic factor and tropomyosin-related kinase B signaling pathway [30,31]. These studies have laid a certain foundation for the application of DFV in treating depression. To explore the antidepressant targets and related molecular regulatory mechanisms of DFV, this study combined systemic pharmacology and in vitro experiments to carry out related work. An LPS-induced BV-2 cells model was applied to assess the effects of DFV on protein regulation and oxidative stress-related processes. These results could provide innovative ideas and evidence for DFV to treat depression. The framework of this research is shown in Figure 1.

## 2. Material and Methods

### 2.1. Retrieval and Screening of Active Compounds in Polygonati Rhizoma

All the compounds in PR were obtained from the Traditional Chinese Medicine Systems Pharmacology Database and Analysis Platform (TCMSP, https://tcmsp-e.com/, accessed on 5 November 2022) [32]. For drugs that play a role in the systema nervosum centrale, with the exception of common drug active parameter settings, the blood–brain barrier (BBB) permeability plays a crucial role [33]. This parameter is important to assess the ability of a compound to enter the central nervous system. The BBB is a highly specific vascular interface that maintains brain homeostasis by separating the blood chamber from the central nervous system. The value of BBB ≥ −0.3 was considered to have a moderate-to-strong degree of central penetration [34]. As stated by the recommended conventional ADME parameter (absorption, distribution, metabolism, and excretion), oral bioavailability (OB) is used as a significant pointer to identify the characteristics of bioactive molecules for oral administration, and the screening critical condition was set to OB ≥ 30% [35]. The “drug-like” (DL) level of a compound greater than or equal to 0.18 was the selection criteria for “drug-like” compounds in traditional Chinese medicine [36]. The half life (HL) of a drug determines the time scale at which the compound may trigger treatment. The screening critical condition of HL was set to be not less than 4 h [37].

### 2.2. Target Acquisition of Candidate Compounds in Polygonati Rhizoma

The targets of all candidate compounds screened by ADME were gained from the TCMSP and Drugbank database (https://go.drugbank.com/, accessed on 5 November 2022). It was standardized and converted by the Uniprot database (https://www.uniprot.org/, last accessed on 10 November 2022) for the gene symbol of protein targets [38].

### 2.3. Acquisition of Depression-Related Targets

The keyword “Depression” was entered, respectively, in the PharmGKB database (https://www.pharmgkb.org/, accessed on 9 November 2022) [39], the Therapeutic Target Database (TTD, http://db.idrblab.net/ttd/, accessed on 9 November 2022) [40], the Online Mendelian Inheritance in Man database (OMIM, https://www.omim.org/, accessed on 9 November 2022) [41], the Genetic Association Database (GAD, https://geneticassociationdb.nih.gov/, accessed on 5 November 2022) [42], and the DisGeNET database (https://www.disgenet.org/, accessed on 5 November 2022) to obtain targets related to depression [43]. Genes from different databases were compared, deleting duplicates to obtain all relevant targets for depression.

### 2.4. Networks Construction

Cytoscape3.8.0 software was used to draw the PR candidate compounds–targets network, depression-related targets network, and PR candidate compounds–depression targets network. The topology parameters of these networks were obtained by using the network-analysis plug-in in the software [44].

### 2.5. Enrichment Analysis

The Database for Annotation, Visualization, and Integrated Discovery (DAVID, https://david.ncifcrf.gov/, accessed on 11 November 2022) was applied to analyze the enrichment results of the targets of PR in treating depression, including gene ontology (GO) analysis and KEGG analysis. GO analysis has the function of unifying the expression of genes and gene-product attributes of all species, including biological processes (BP), cellular components (CC), and molecular functions (MF) [45]. KEGG is a knowledge base for the biological interpretation of fully sequenced genomes through pathway mapping [46]. KEGG pathways, GO-MF, GO-BP, and GO-CC were selected for analysis in the enrichment section. The entries were selected for further analysis when their feature annotation with a significant *p*-value (corrected by Bonferroni and Benjamini algorithms, *p* < 0.05) [47].

### 2.6. Molecular Docking

The 3D structures of DFV were downloaded from the Pubchem platform (https://pubchem.ncbi.nlm.nih.gov/compound/114829, accessed on 12 November 2022) as a ligand. The crystal structure of cyclooxygenase 2 (COX2, PDB ID: 5IKR) was obtained from the PDB platform (https://www.rcsb.org/structure/5IKR, accessed on 12 November 2022) as a receptor [48]. We used the CB-Dock (https://cadd.labshare.cn/cb-dock/php/blinddock.php, accessed on 12 November 2022) database platform to execute the online-docking program [49], with a grid-box size of (X: 31.8585, Y: 27.2645, Z: 71.9915). Their binding affinity was estimated based on the Vina score [50].

### 2.7. Reagents

BV-2 cells were obtained from Fuheng Biology (Shanghai, China). DFV (Liquiditigenin) was purchased from MCE (Shanghai, China). LPS (Mutant of *E. coli* O111: B4.) was provided by Sigma Aldrich (St. Louis, MO, USA). Dulbecco modified Eagle medium (DMEM) and fetal bovine serum (FBS) were provided by Gibco (Grand Island, NE, USA); 0.25% trypsin and penicillin streptomycin were obtained from Beyotime (Beijing, China). Dimethyl sulfoxide (DMSO) was purchased from Abcam (Shanghai, China). The Superoxide dismutase (SOD) and malondialdehyde (MDA) test kits, cell counting kit-8 (CCK-8), BCA Protein Assay Kit, and SDS-PAGE gel preparation kit were provided by Beyotime (Beijing, China).

### 2.8. CCK8 Assay

Cell counting kit-8 was used to evaluate the viability of BV-2 cells. The cell concentration of 3 × 10^3^ cells/well was inoculated into a 96-well plate. One hundred μL of medium containing the corresponding concentration of drugs were added when the cells adhered to the wall and fused to 70–80% and were incubated for a certain time. Then, 10 μL CCK-8 were added to each hole. After 1 h of incubation, the optical density (OD) was measured with a Spectra MAX iD3^®^ Multi-mode microplate reader (Sunnyvale, CA, USA) at 450 nm wavelength. Cell viability was calculated as cell viability = (OD (treatment group) − OD (blank))/(OD (control group) − OD (blank)) × 100%.

### 2.9. SOD and MDA Detection

BV-2 cells were tiled on 6-well plates. After 12 h culture in the cell incubator, BV-2 cells were preincubated with DFV (5, 10, 25 μM) for 1 h and then treated with LPS (2 μg/mL) for 24 h. After treatment, the cells were washed with PBS twice, and 150 μL of RIPA lysate were added to each well to fully lysate the cells. After centrifugation at 4 °C at about 12,000× *g* for 10 min, the supernatant was taken as the sample to be tested. The protein concentration of each sample was quantified by the BCA kit. Then, the SOD and MDA levels in the supernatant were detected. Specific operations were carried out according to the operating instructions of the BCA protein-concentration assay kit, total SOD activity assay kit, and MDA Assay Kit (Beyotime, Beijing, China) [51]. The OD of each hole was measured with a Spectra MAX iD3^®^ multi-mode microplate reader (Molecular Devices, Sunnyvale, CA, USA). The absorbance of SOD activity was measured at 450 nm, and that of MDA content was measured at 532 nm. The BCA was used to calculate the protein concentration by determining the absorbance value at 562 nm.

### 2.10. Quantitative Real-Time PCR Assay

Total RNA was extracted from the BV-2 cells of different treatment groups using the EZ-Press RNA purification kit (EZ Bioscience, Roseville, CA, USA). Then, a Reverse Transcription Kit (EZ Bioscience, Roseville, CA, USA) was used to convert the extracted RNA into cDNA using specific primers (EZ Bioscience, Roseville, CA, USA) to amplify the cDNA templates. The mRNA levels of various genes (COX2, TNF-α, IL-1β, and IL-6) compared with GAPDH were evaluated by 2^−ΔΔCt^. The primer sequences used in this study refer to previous studies [52,53,54] (Table 1).

### 2.11. Morphologic Observation of BV-2 Cells and Rhodamine123 Staining

The cells were spread in 6-well plates containing slides and grouped and treated in the same way as before. After the treatment, the 6-well plates containing slides were rinsed with PBS three times. The cells were marked with Rhodamine123 (Beyotime, Shanghai, China) in a dark environment for a few minutes [55]. The dye solution in the hole was discarded, and the 6-well plate was washed with PBS three times. The slide was taken out and placed on a glass support containing an anti-fluorescence quenching agent (Beyotime, Shanghai, China) and photographed under a fluorescence microscope (Olympus, Tokyo, Japan).

### 2.12. Western Blot Analysis

After different treatments, the BV-2 cells were washed with cold PBS twice, and the cells were fully lysed with RIPA lysate containing 1% protease inhibitor. The lysate was collected and centrifuged at 4 °C at 12,000 rpm for 25 min. Part of the supernatant was taken for protein-concentration determination, and the remaining supernatant was mixed with the loading buffer and boiled at 100 °C for 10 min. Fifty μg of protein were injected into the pore of 12% and 8% SDS polyacrylamide gel to isolate proteins with different molecular weights. The protein was then transferred at a constant voltage to a PVDF membrane (Millipore, Bedford, MA, USA). The membrane was immersed in TBST solution containing 5% skim milk and slowly shaken at room temperature for 1 h. Wash the protein band with TBST three times. Anti-β-actin (1:1000) (Affinity, Changzhou, China), Anti-COX2 (1:1000) (Bioss, Beijing, China), anti-NLRP3 (1:1000) (GeneTex, Southern California, CA, USA), and anti-Caspase-1 (1:500) (Wanleibio, Shenyang, China) antibodies were used to incubate the protein bands at 4 °C overnight. On the second day, protein bands were incubated with either goat anti-rabbit (1:10,000) or goat anti-mouse secondary antibody (1:10,000) (ZSGB BIO, Beijing, China) for 1 h. All protein bands were measured by ECL Western blot assay.

### 2.13. Statistical Analysis

The results of the in vitro verification experiment were obtained by three repeated experiments, expressed as mean ± SEM. For the comparison of more than two groups, a one-way analysis of variance (ANOVA) and an unpaired *t*-test were used with GraphPad prism software(version 9). It was considered that *p* < 0.05 was a significant difference between the two groups, and *p* < 0.01 was extremely significant.

## 3. Results

### 3.1. Acquisition of Candidate Compounds and Targets in Polygonati Rhizoma

A total of 38 compounds in PR were obtained from the TCMSP database. The detailed information about the ADME parameters of the compounds is provided in Supplementary Table S1. The parameter-screening conditions of the active candidate compounds were set as BBB ≥ −0.3, OB ≥ 30%, DL ≥ 0.18, and HL ≥ 4. A total of seven candidate compounds were obtained (Table 2).

These compounds included flavonoids such as DFV (MOL001792), baicalein (MOL002714), (2R)-7-hydroxy-2-(4-hydroxyphenyl) chroman-4-one (MOL004941) and 4′,5-Dihydroxyflavone (MOL006331). Beta-sitosterol (MOL000358) and sitosterol (MOL000359) were classified as sterol compounds. Diosgenin (MOL000546) belongs to saponins.

**Figure 2:**
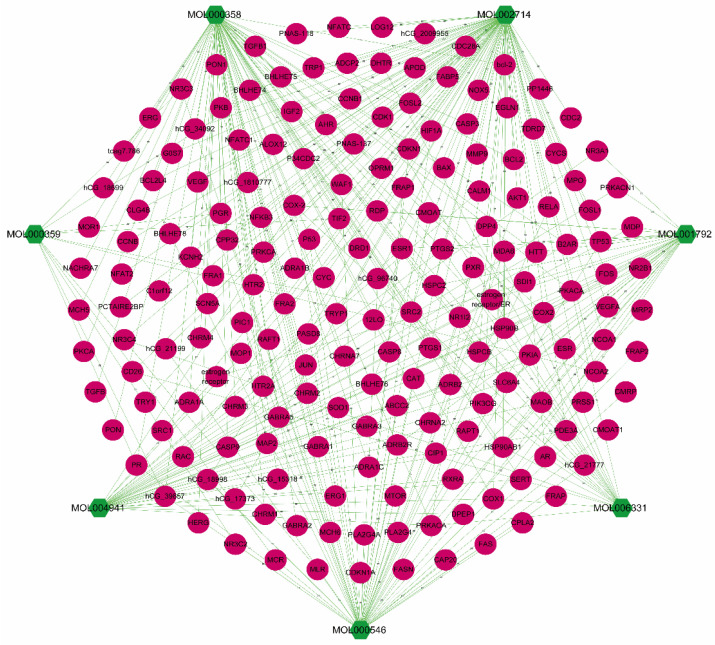
PR candidate compounds–targets network. Cytoscape 3.8.0 software was used to construct the association between PR and candidate targets, and different shapes and colors were used to render the compound and target features represented by nodes in the network for differentiation. The green hexagonal nodes represented candidate compounds in PR, the rose-red circular nodes represented the matching targets of the compounds, and the edges represented association relationships between nodes.

The information on targets corresponding to the seven candidate compounds was excavated from the TCMSP and the Drugbank database (Supplementary Table S2). MOL001792 corresponded to 12 target proteins and 31 genes. MOL002714 corresponded to 37 target proteins and 94 genes. MOL000358 corresponded to 38 target proteins and 73 genes. MOL000359 corresponded to three target proteins and 11 genes. MOL0004941 corresponded to 15 target proteins and 34 genes. MOL000546 corresponded to 16 target proteins and 55 genes. MOL006331 corresponded to eight target proteins and 19 genes. The candidate compounds–targets network of PR is shown in Figure 2. The network contained 194 nodes and 317 edges, of which 7 nodes were compounds and 187 nodes were their targets.

### 3.2. Targets Collection for Depression

**Figure 3:**
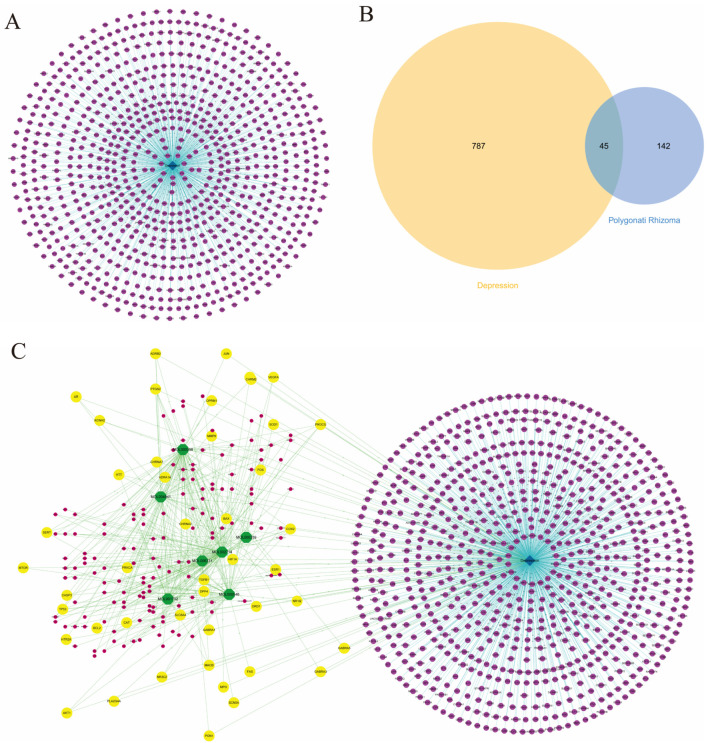
The construction of depression targets network and interacting with PR-related targets. (**A**) Depression-targets network. The blue diamond nodes represented depression, while the purple circular nodes represented depression-related targets. (**B**) The common targets between PR and depression are shown in the Venn diagram. (**C**) PR candidate compounds–targets–depression targets joint network. The yellow nodes represented common targets for PR and depression. The rose-red nodes represented other targets in PR that were not related to depression. Purple nodes represented depression targets unrelated to PR. Green nodes represented candidate compounds of PR. The blue diamond node represented depression.

By inputting the keyword “depression”, 23 genes were obtained from the PharmGKB database, 76 genes were collected from the OMIM database, 683 genes were gathered from the DisGeNET database, 43 genes were obtained from the TTD database, and 185 genes were obtained from the GAD database. After merging the same genes, a great sum of 832 depression-related genes was obtained (Supplementary Table S3), and then, the depression-targets network was constructed (Figure 3A). By comparing PR targets and depression targets, we found 45 potential targets for PR intervention in depression (Figure 3B). After integrating the candidate compounds–targets network of PR with the depression-targets network, we obtained the PR candidate compounds–targets–depression targets joint network (Figure 3C).

### 3.3. PR–Depression Targets Network Construction and Enrichment Analysis

**Figure 4:**
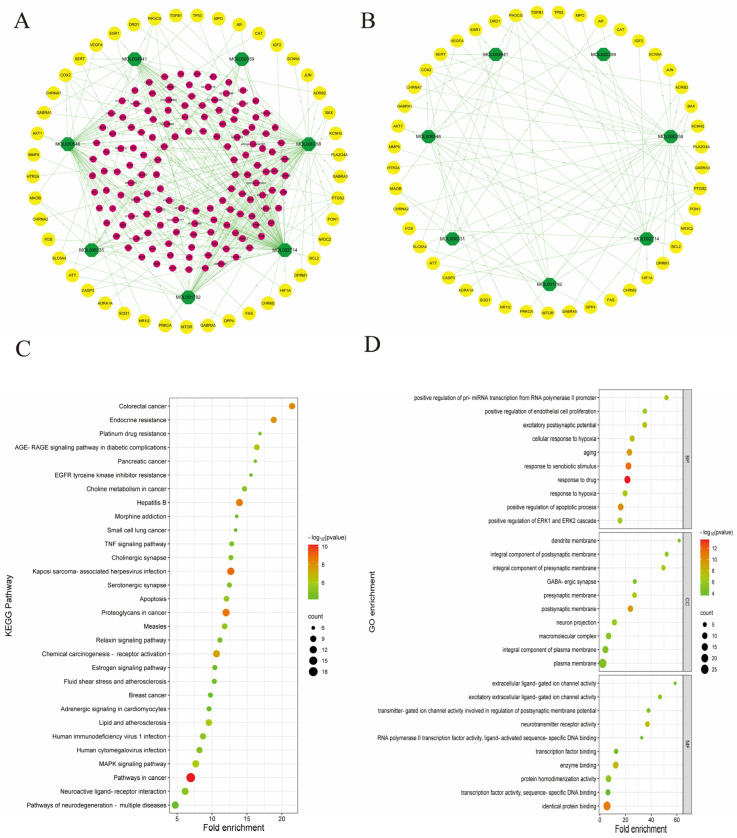
Construction of PR candidate compound−depression targets network and enrichment analysis of PR antidepressant targets. (**A**) PR candidate compounds−depression targets−other targets network. (**B**) PR candidate compounds−depression targets network (**C**) Advanced bubble map of KEGG enrichment analysis of targets. (**D**) Advanced bubble chart of the top-ten entries in BP, CC, and MF of target GO enrichment analysis.

First, depression targets unrelated to PR were removed (Figure 4A). Ultimately, by removing PR targets that are not associated with depression, the candidate compounds–depression targets a network of PR was obtained (Figure 4B). By enrichment analysis of genes in the network, these genes were involved in pathways in cancer, neuroactive ligand–receptor interaction, Kaposi sarcoma-associated herpesvirus infection, pathways of neurodegeneration−multiple diseases, etc. (Supplementary Table S4). The first 30 entries in the result list were shown based on the *p*-value (Figure 4C). Similarly, we found that these genes were involved in BP, including response to a drug, response to xenobiotic stimulus, have positive regulation of the apoptotic process, response to lipopolysaccharide, etc. Specific information on each item is displayed in Supplementary Table S5, and Figure 4D shows the top-10 entries according to *p*-value. In terms of CC, these genes participated in the postsynaptic membrane, an integral component of the presynaptic membrane, presynaptic membrane, neuron projection, etc. (Supplementary Table S6, Figure 4D). With respect to MF, these genes were associated with identical protein binding, enzyme binding, neurotransmitter receptor activity, protein homodimerization activity, and so on (Supplementary Table S7, Figure 4D).

### 3.4. Molecular Docking of COX2 with Correlative Compounds

**Figure 5:**
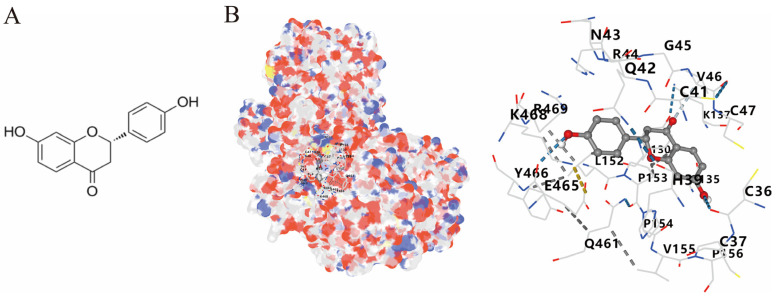
Molecular docking between DFV and COX2. (**A**) Chemical structure of DFV. (**B**) Docking conformation of DFV and COX2 (PDB ID: 5IKR) and amino acid residues at the binding site.

After analyzing the topological features of the nodes in the PR–depression targets network, it was found that COX2 has a more significant degree value (6) in the target nodes (Supplementary Table S8). On the whole of COX2-related compounds, DFV was selected as the intervention drug for subsequent experimental verification. Prior to this, we first observed the binding conformation between the ligand and the receptor. The structure of DFV is shown in Figure 5A. Then, we performed molecular docking of DFV with COX2 (PDB: 5IKR), and the binding conformation is shown in Figure 5B. The docking result showed that the Vina score for its docking score was −8.9 kcal/mol. The amino acid residues surrounding the compound were CYS36, CYS37, HIS39, CYS41, ARG44, GLY45, VAL46, CYS47, TYR130, GLY135, LEU152, PRO153, PRO154, PRO156, GLN461, GLU465, TYR466, LYS468, and ARG469.

### 3.5. Effect of DFV on Expression of Inflammatory Cytokines in LPS-Induced BV-2 Cells

**Figure 6:**
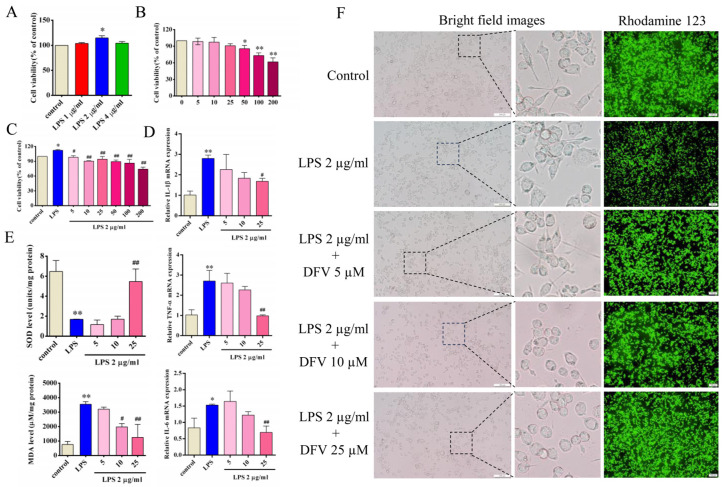
The effect of DFV on oxidative stress and inflammatory response in LPS-induced BV-2 cells. (**A**) LPS enhances the cell viability of BV-2 cells. (**B**) Effect of DFV on BV-2 cell viability. (**C**) DFV inhibited the viability of BV-2 cells in a culture system containing 2 μg/mL LPS. (**D**) DFV regulated mRNA expression levels of IL-1β, TNF-α, and IL-6 in LPS-induced BV-2 cells. (**E**) DFV improved SOD and reduced MDA production in LPS-induced BV2 cells. (**F**) DFV inhibits amoeba-like morphological changes and increases mitochondrial membrane potential in LPS-induced BV-2 cells. Data are rendered as mean ± SEM. Compared with the control group, * *p* < 0.05, ** *p* < 0.01. Compared with the LPS group, ^#^ *p* < 0.05, ^##^ *p* < 0.01.

We first investigated the effects of LPS on the viability of BV-2 cells at different concentrations. The results of the CCK8 assay showed that the viability of BV-2 cells was enhanced when LPS concentration was 2 μg/mL (*p* < 0.05) (Figure 6A). Subsequently, we determined the toxicity of DFV to BV-2 cells at different concentrations. There was insignificant cytotoxicity of DFV to BV-2 cells in the concentration range of 0–50 μM (Figure 6B). Further, we determined the effects of different concentrations of DFV on a BV-2 cell-culture system containing 2 μg/mL LPS. The results showed that 5 μM DFV significantly inhibited enhanced BV2 cell viability induced by LPS (*p* < 0.05) (Figure 6C). By determining the gene expression of several inflammatory cytokines in different treatment groups, it was found that the expressions of IL-1β, TNF-α, and IL-6 in BV-2 cells were significantly increased after LPS induction compared with the control group (*p* < 0.05). However, pre-incubation with DFV in a culture system containing LPS, mRNA levels of IL-1β, TNF-α, and IL-6 in BV-2 cells were reduced dose dependently compared with those in the LPS group. Moreover, when the concentration of DFV was 25 μM, the decrease was statistically significant (*p* < 0.05) (Figure 6D).

### 3.6. Effect of DFV on MDA and SOD Release in LPS-Induced BV-2 Cells

By determining the MDA and SOD levels in BV-2 cells, LPS (2 μg/mL) significantly increased the production of MDA and reduced the level of SOD in BV-2 cells. Cells pretreated with DFV showed a dose-dependent inhibition of MDA and an increase of SOD and showed a strong ability to reverse the LPS effect when the DFV concentration was 25 μM (*p* < 0.01) (Figure 6E).

### 3.7. Effect of DFV on Mitochondrial Membrane Potential of LPS-Induced BV-2 Cells

Through the same above treatment, the cells were added to LPS and photographed under the microscope 24 h later. It was found that BV-2 cells were activated in the culture system containing 2 μg/mL, showing an amoeba-like appearance. BV-2 cells pretreated by DFV showed insignificant activation after LPS induction (Figure 6F). Subsequently, the cells were stained with rhodamine123. The results showed that, compared with the control group, the LPS group significantly decreased the mitochondrial membrane potential and fluorescence intensity due to the activation and inflammatory response of BV-2 cells. Compared with the LPS group, the mitochondrial fluorescence of the BV-2 cells pretreated with DFV was prominently enhanced (Figure 6F).

### 3.8. Effects of DFV on the Expression of COX2, NLRP3, and Caspase1 in LPS-Induced BV-2 Cells

**Figure 7:**
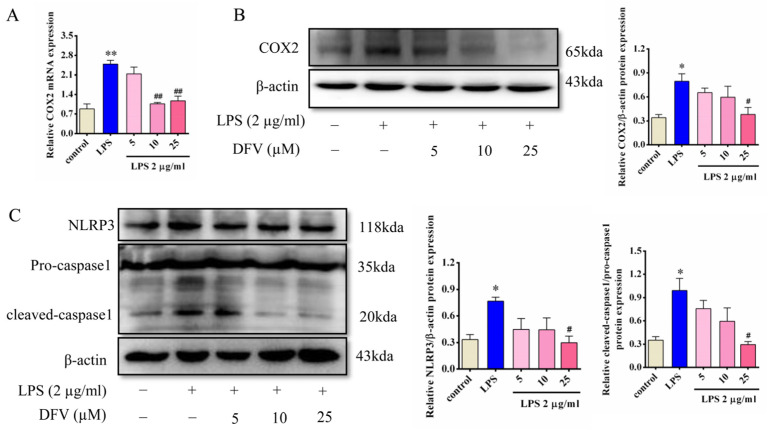
The effect of DFV on the expression of COX2 and inflammatory-related proteins in LPS-induced BV-2 cells. (**A**) DFV inhibited an increased level of COX2 mRNA in LPS-induced BV-2 cells. (**B**) DFV inhibited increased protein an level of COX2 induced by LPS in BV-2 cells. (**C**) DFV inhibited increased NLRP3 and cleaved caspase1 protein levels in LPS-induced BV-2 cells. Data are rendered as mean ± SEM. Compared with the control group, * *p* < 0.05, ** *p* < 0.01. Compared with the LPS group, ^#^ *p* < 0.05, ^##^ *p* < 0.01.

Molecular docking showed that DFV had good affinity and stable conformation with COX2. The results of qPCR and Western blot showed that the expression levels of the COX2 gene and protein in BV-2 cells were significantly increased when LPS existed in the system. The gene and protein levels of COX2 in DFV-pretreated cells were inconspicuously increased, while they were significantly decreased compared with the LPS group. COX2 mRNA expression significantly decreased at DFV 10 μM (Figure 7A), and COX2 protein expression significantly decreased at DFV 25 μM (*p* < 0.05) (Figure 7B). It indicated that the effect was dose dependent. At the same time, for NLRP3 and caspase1, as downstream inflammatory proteins closely related to COX2, as expected, the expression levels of NLPR3 and cleaved-caspase1 were apparently increased in LPS-induced BV-2 cells, while the level of caspase1 remained unchanged in all groups. Expression levels of NLPR3 and cleaved caspase1 were markedly lower in DFV-pretreated cells than in the LPS group dose-dependently. The effects had statistical significance at a DFV concentration of 25 μM (*p* < 0.05) (Figure 7C).

## 4. Discussion

Traditional Chinese Medicinal herbs of the genus Polygonatum have been used as a tonic in China, India, Pakistan, Iran, and Japan [56]. The crude extract and some pure compounds of PR have many pharmacological effects, such as anti-aging, anti-diabetes, anti-fatigue, and anti-cancer [57]. The neuroprotective effects of PR have also received attention. Studies have shown that PR has pharmacological effects against AD with the mechanism of preventing deposition of the Aβ protein [20], improving learning and memory function, and reducing neuronal apoptosis [56].

Similarly, depression is a mental disorder with several neurodegenerative pathophysiological mechanisms that are associated with neuroinflammatory activity in response to external stimuli in specific areas of the brain. Therefore, intervening in neuroinflammatory processes is also considered an effective means of fighting depression [58,59]. LPS-induced astrocyte activation is a commonly used in vitro model of neuroinflammation, simulating stress stimuli in the central nervous system. The immune response of the central nervous system stimulated by stress is characterized by rapid activation of microglia and astrocytes and proinflammatory cytokines. This process includes the release of IL-1 β, TNF-α, and prostaglandin E2 (PGE2). Excessive COX2 enzyme activity can promote the production of PGE2, a pro-inflammatory factor [60]. In the LPS-induced depression mouse model, the transcription function of the CREB/ATF-2 family is upregulated, leading to an overexpression of COX2. This induces excessive activity in the inflammatory response, leading to behavioral depression in mice [61]. As a key protein in the pathogenesis of neuroinflammation, COX2 is increasingly valued for its role in the transmission and cascade amplification of inflammation. Among them, the p38 MAPK/ATF-2 signaling pathway has been shown to play a role in inducing COX2-derived PGE2 production in inflammation in brain injury and inflammatory diseases [62]. Currently, many studies have also shown that the activation of COX2 is an important factor mediating the development of depression [63,64]. COX2 produces prostaglandin-endoperoxide synthase (PTGS) under the regulation of specific stimulus events, such as responding to physiological stress responses, infection, and inflammation. Usually, COX2 and PTGS2 are combined to represent [65,66,67,68]. In this study, after conducting network-feature topology analysis on the PR candidate compounds–depression targets network, it was found that COX2 and PTGS2 have the same degree value, which is the largest value among the targets. Therefore, we selected COX2 as the core target in our study. The research results found that six candidate compounds in PR are directly related to COX2. PR is rich in various active ingredients, including polysaccharides, saponins, and flavonoids. Flavonoids are important secondary metabolites of PR and vital indicators for measuring the quality of PR from various habitats [69]. Studies have shown that the content of DFV in the same lifetime of PR is lower than β-sitosterol but higher than other active compounds [70]. β-sitosterol has been proven to exist as a potential active ingredient in antidepressant formulations in multiple network pharmacology studies [71,72]. Therefore, this study selected DFV with a smaller molecular weight (MW: 256.27) as the compound to be validated. DFV is a flavonoid compound widely present in Chinese herbal medicines such as PR, Glycyrrhizae radix et rhizome (Pharmacopoeia of China, 2015), Isatidis folium (Pharmacopoeia of China, 2015), Hedysari Radix (Pharmacopoeia of China, 2015), Epimedii folium (Pharmacopoeia of China, 2015), etc. The liver- and heart-protective effects of DFV have been extensively studied [26,73,74,75]. In addition, there have been some reports on the neuroprotective effects of DFV that we are concerned about. DFV also exhibits a protective effect on glutamate-mediated neuronal cell death [76]. Similarly, DFV also has neuroprotective effects on Aβ (25–35)-induced neurotoxicity [77]. In this study, we first predicted the molecular docking conformation of DFV and COX2 and found that they have good affinity. It is generally believed that, when the absolute value of the molecular docking score is greater than seven, small molecules and proteins are more likely to bind [78,79]. We found and demonstrated that COX2 can be an effective target for DFV in antidepressant therapy. We also affirmed that the mRNA and protein expressions of COX2 were increased by LPS in BV-2 cells. It leads to the upregulation of NLRP3 and promotes caspase1 cleavage. This process triggers the release of inflammatory factors in cells, such as TNF-α, IL-6, and IL-1β. This may be an important reason for the activation of BV-2 cells and the occurrence of amoeba-like morphological changes, while DFV reversed these effects of LPS. This may be one of the molecular regulatory mechanisms for its anti-depression effect.

## 5. Conclusions

**Figure 8:**
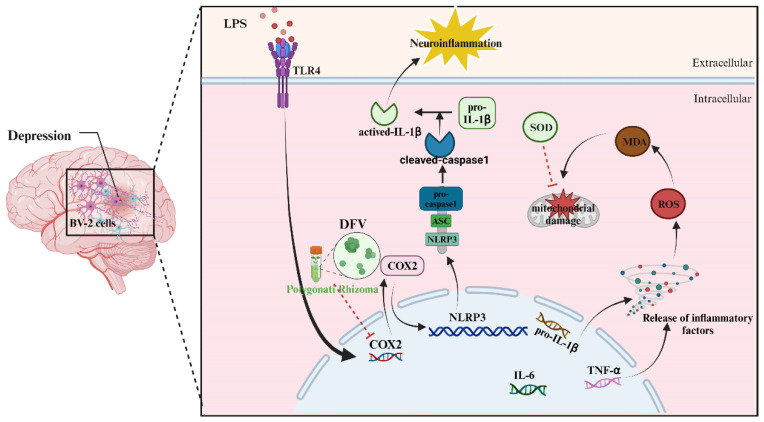
The antidepressant mechanism of the Polygonati Rhizoma extract DFV in a BV-2 cell model by inhibiting COX2-mediated inflammatory response (created with BioRender.com, accessed on 12 March 2024). In BV-2 cells, LPS promotes the gene expression and protein translation of COX2 by binding to receptors, further promoting the gene and protein expression of NLRP3, leading to the release of inflammatory factors such as IL-1 β, IL-6, and TNF-α. The inflammatory environment causes the accumulation of reactive oxygen species (ROS), which damages mitochondria, manifested as a decrease in mitochondrial membrane potential. ROS promotes the accumulation of oxidative product MDA, which exacerbates mitochondrial damage. SOD prevents and repairs this damage process. On the other hand, NLRP3 promotes the hydrolysis and cleavage of caspase1, further inducing the release of activated IL-1 β and triggering neuroinflammation. The compound DFV in Polygonati Rhizoma reduced the gene expression and protein translation of COX2 by binding to it, thereby preventing subsequent inflammatory reactions.

In conclusion, we used systemic pharmacology to screen active compounds in Polygonati Rhizoma and their anti-depression targets primitively and found that DFV may be an important component of PR in exerting antidepressant effects. Molecular docking preliminarily confirmed the binding affinity between DFV and COX2. In vitro, DFV reversed the overexpression of MDA and inhibition of SOD expression in LPS-induced BV-2 cells. Further, DFV was found to inhibit COX2 overexpression induced by LPS, which further inhibited the transitional activation of NLRP3 and caspase1 in BV-2 cells. This was manifested by the reduced release of inflammatory cytokines, such as TNF-α, IL-6, and IL-1β, and the recovery of reduced mitochondrial membrane potential (Figure 8). These results proved the compound basis and pharmacological mechanism of the anti-depression of Polygonati Rhizoma. However, we must also declare that the work of this study still has limitations in the construction of animal models, confirmation of the efficacy of DFV in vivo, and validation of its pharmacological mechanisms. We will conduct more in-depth research on these aspects in our current and future work.

## Figures and Tables

**Table 1:** The primer sequences of COX2, TNF-α, IL-6, IL-1β, and GAPDH.

Gene Name	Sequences
COX2	(F) 5′-TGAGCATCTACGGTTTGCTG-3′ (R) 5′-TGCTTGTCTGGAACAACTGC-3′
TNF-α	(F) 5′-AAAATTCGAGTGACAAGCCTGTAG-3′ (R) 5′-CCCTTGAAGAGA-ACCTGGGAGTAG-3′
IL-6	(F) 5′-AGATACAAAGAAATGATGGATGCTA-3′ (R) 5′-TCTTGGTTGAAGATATGAATTAGAG-3′
IL-1β	(F) 5′-GTGTCTTTCCCGTGGACCTT-3′ (R) 5′-CGTTGCTTGGTTCTCCTTG-3′
GAPDH	(F) 5′-TAGATTATTCTCTGATTTGGTCGTATTGG-3′ (R) 5′-GCTCCTGGAAGATGGTGATGG-3′

**Table 2:** Parameter information of candidate compounds.

Mol ID	Molecular Name	OB (%)	DL	BBB	HL	Structure
MOL001792	DFV	32.76	0.18	−0.29	17.89	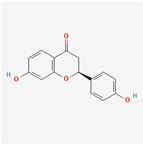
MOL002714	baicalein	33.52	0.21	−0.05	16.25	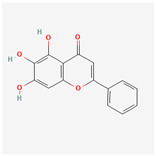
MOL000358	beta-sitosterol	36.91	0.75	0.99	5.36	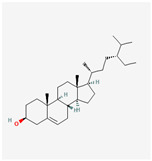
MOL000359	sitosterol	36.91	0.75	0.87	5.37	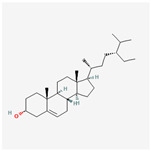
MOL004941	(2R)-7-hydroxy-2-(4-hydroxyphenyl) chroman-4-one	71.12	0.18	−0.25	18.09	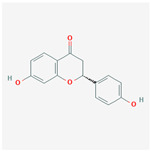
MOL000546	diosgenin	80.88	0.81	0.27	4.14	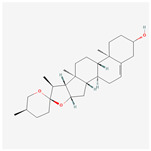
MOL006331	4′,5-Dihydroxyflavone	48.55	0.19	−0.03	18.01	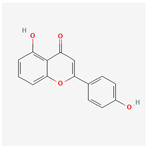

## Data Availability

The original data involved in the study are included in the article/Supplementary Material. Further inquiries can be directed to the corresponding author.

## Supplementary Materials

The following supporting information can be downloaded at: https://www.mdpi.com/article/10.3390/nu16081167/s1, Table S1: Compounds of Polygonati Rhizoma. Table S2: Targets information of candidate compounds. Table S3: Depression-related genes from different databases. Table S4: Information of KEGG pathway. Table S5: Information of biological processes. Table S6: Information of cellular components. Table S7: Information of molecular functions. Table S8: Topological information of the PR candidate compounds-depression targets network.

## Abbreviations

AD: Alzheimer’s disease; ADME: Absorption, Distribution, Metabolism, Excretion; BBB: blood-brain barrier; BP: biological processes; CC: cellular components; CCK8: cell counting kit-8; COX2: cyclooxygenase 2; DAVID: Database for Annotation, Visualization, and Integrated Discovery; DL: drug-like; DMEM: Dulbecco modified Eagle medium; DMSO: Dimethyl sulfoxide; FBS: fetal bovine serum; GAD: Genetic Association Database; GO: Gene Ontology; HL: half-life; IL-1β: interleukin-1β; LPS: Lipopolysaccharide; MDA: malondialdehyde; MF: molecular functions; NLRP3: NACHT, LRR and PYD domains-containing protein 3; OB: Oral bioavailability; OD: optical density; OMIM: Online Mendelian Inheritance in Man database; PGE2: prostaglandin E2; PR: Polygonati Rhizoma; PTGS: prostaglandin-endoperoxide synthase; ROS: reactive oxygen species; SOD: Superoxide dismutase; TCM: traditional Chinese medicine; TCMSP: Traditional Chinese Medicine Systems Pharmacology Database and Analysis Platform; TNF-α: tumor necrosis factor-α; TTD: Therapeutic Target Database; 5-HT: 5-hydroxytryptamine.
